# Multimodal brain network disruption and structural-functional decoupling in overt hypothyroidism

**DOI:** 10.3389/fendo.2026.1763670

**Published:** 2026-04-17

**Authors:** Hong Yu, Xue-Huan Liu, Xiao Gao, Yu-Ting Wang, Fei-Fei Zheng, Zhi-Bo Zhou, Gouling Zhan, Weiwei Cui, Xiao-Han Zheng, Hai-Lin Shao, Hao Wang, Qing He, Jun Liu

**Affiliations:** 1 Department of Radiology, The Fourth Central Hospital Affiliated to Tianjin Medical University, Tianjin, China; 2 Department of Radiology, Tianjin Union Medical Center, The First Affiliated Hospital of Nankai University, Nankai University, Tianjin, China; 3 Department of Endocrinology, The Fourth Central Hospital Affiliated to Tianjin Medical University, Tianjin, China; 4 Department of Endocrinology and Metabolism, Tianjin Medical University General Hospital, Tianjin, China

**Keywords:** diffusion tensor imaging, graph theory, hypothyroidism, resting-state functional MRI, structural-functional coupling

## Abstract

**Background:**

Overt hypothyroidism (OH) is frequently associated with cognitive impairment and emotional disturbances, yet its impact on the fundamental relationship between brain structure and function remains unclear.

**Methods:**

A total of 45 patients with OH and 55 healthy controls (HCs) were enrolled in this study. All participants underwent psychological scale assessments, as well as diffusion tensor imaging (DTI) and resting-state functional magnetic resonance imaging (rs-fMRI) scans. Based on the AAL90 atlas, structural connectivity (SC) was constructed from DTI data using PANDA, while functional connectivity (FC) was derived from rs-fMRI data using DPABI. Global and nodal topological properties were calculated using GRETNA and compared between groups. Whole-brain SC-FC coupling was computed as the Pearson correlation between non-zero SC edges and their corresponding FC values. Cortical regions were divided into seven Yeo-7 networks, with subcortical structures as a separate group, to calculate SC-FC coupling within each subnetwork. All results were FDR-corrected for multiple comparisons.

**Results:**

Compared with HCs, the OH group showed reduced small-worldness and clustering coefficient in structural networks, alongside decreased global efficiency in both structural and functional networks. Nodal analyses revealed reduced efficiency and centrality in multiple key regions, including nodes within the default mode network, limbic system, and salience network. Notably, OH patients displayed significantly weakened SC-FC coupling within the limbic system and salience networks. The disruption of these brain networks was significantly associated with both poorer cognitive performance and emotional disturbances in OH.

**Conclusions:**

This study demonstrates topological disruptions and impaired SC-FC coupling in specific brain networks in OH, providing new insights into the neurobiological mechanisms underlying cognitive and emotional deficits.

## 1 Introduction

Overt hypothyroidism (OH) is a common endocrine disorder and is frequently accompanied by cognitive impairment and affective symptoms (1). Thyroid hormones play a critical role in central nervous system function throughout life, supporting neurodevelopment and, in adulthood, maintaining energy metabolism, neurotransmitter homeostasis, and myelination (2). Consistent with these roles, patients with hypothyroidism often exhibit neuropsychiatric symptoms such as apathy, depressed mood, and cognitive slowing, suggesting system-level alterations in brain structure and function (3, 4).

Neuroimaging studies have provided converging evidence of brain abnormalities in hypothyroidism. Structural magnetic resonance imaging (MRI) has revealed gray matter reductions in regions involved in cognitive and emotional regulation, including the prefrontal cortex and medial temporal lobe (5, 6). Functional MRI (fMRI) studies have reported decreased functional connectivity (FC) within the right frontoparietal attention network and the somatomotor network, alongside increased connectivity within the default mode network (DMN) (7). Altered connectivity has also been observed between the cerebellum and frontal and parietal regions, as well as between the hippocampus and widespread cortical areas (8). Diffusion tensor imaging (DTI) further demonstrates widespread microstructural abnormalities in white matter (9, 10). In addition, magnetic resonance spectroscopy has identified regional metabolic alterations, including reduced γ-aminobutyric acid levels in the medial prefrontal cortex and elevated choline-containing compounds in the dorsolateral prefrontal cortex and posterior parietal cortex (11). However, these findings are largely derived from single-modality analyses and do not directly address the organization of whole-brain networks.

With advances in neuroimaging, multimodal MRI enables the investigation of structural and functional brain alterations at the network level. DTI can be used to characterize structural connectivity (SC), whereas resting-state fMRI (rs-fMRI) assesses FC between brain regions (12). Graph theory provides a quantitative framework for evaluating network organization and information transfer efficiency (13). Within this framework, the brain is modeled as a network composed of nodes (brain regions) and edges (their connections). This connectome typically exhibits small-world properties, balancing local specialization and global integration to support efficient information processing (14). Previous studies applying graph-theoretical analysis to white matter networks in hypothyroidism have reported reduced global efficiency and altered local topology, particularly in the limbic system and DMN (15). However, it remains unclear whether the topological organization of functional networks is altered in patients with OH and how functional networks relate to structural networks.

SC-FC coupling captures the concordance between the brain’s anatomical wiring and its functional dynamics, providing a sensitive marker for subtle pathophysiological alterations (16). Increasing evidence has demonstrated structural and functional alterations across neurological and psychiatric disorders (including stroke, Alzheimer’s disease, multiple sclerosis, and depression) using SC-FC coupling as a unified framework (17–20). This approach facilitates the characterization of network integration and segregation and offers insights into neuroplastic mechanisms. Based on the parcellation scheme proposed by Yeo et al., the cerebral cortex can be divided into seven canonical networks, with subcortical regions treated separately (21). This framework allows the assessment of SC-FC coupling at both whole-brain and subnetwork levels.

We hypothesize that OH disrupts the topological organization of large-scale brain networks and weakens the coupling between functional and SC. In this study, we employed multimodal neuroimaging to (1) characterize topological abnormalities of structural and functional brain networks in hypothyroid patients using graph-theoretical analysis, (2) investigate alterations in SC-FC coupling at both the whole-brain and subnetwork levels, and (3) explore the relationships between these network changes and clinical manifestations. Our findings may identify potential neuroimaging biomarkers based on brain network architecture for assessing brain injury in OH and provide insights into the neural mechanisms underlying cognitive and emotional alterations.

## 2 Methods

### 2.1 Participants

This study enrolled 50 treatment-naïve adults with newly diagnosed OH at Tianjin Fourth Central Hospital between July 2024 and October 2025. Participants were eligible if they (1) were right-handed, (2) had ≥6 years of education, (3) were aged 18−60 years, (4) were newly diagnosed and treatment-naïve, and (5) met the diagnostic criteria of the 2017 Chinese Guidelines for the Diagnosis and Treatment of Adult Hypothyroidism, defined as elevated thyroid-stimulating hormone (TSH) with free thyroxine (FT4) below the reference range. Exclusion criteria included a history of neurological or psychiatric disorders, structural brain lesions, severe white matter hyperintensities or early confluent lesions (Fazekas score ≥2), other endocrine disorders, non-thyroid autoimmune diseases, major cardiovascular or other chronic systemic illnesses, significant visual or hearing impairment, substance abuse, pregnancy or lactation, and contraindications to MRI. Healthy controls (HCs) were required to (1) be right-handed; (2) match the patient group in age, sex, and years of education; and (3) have no history of endocrine, systemic, neurological, or psychiatric disorders. After excluding five patients (three due to incomplete MRI data and two due to excessive head motion), a total of 45 patients with OH and 55 HCs were included in the final analysis. The study was approved by the Medical Research Ethics Committee of Tianjin Fourth Central Hospital (Approval No. SZXLL-2023-KY045), and all participants provided written informed consent. The study was conducted in accordance with the Declaration of Helsinki.

### 2.2 Clinical assessments and neuropsychological evaluation

All participants underwent comprehensive clinical and neuropsychological assessments. Demographic and clinical variables, including sex, age, body mass index (BMI), and years of education, were collected. Fasting venous blood samples were obtained to assess thyroid function, including TSH, free triiodothyronine (FT3), FT4, thyroglobulin antibody (TgAb), and thyroid peroxidase antibody (TPOAb). Thyroid hormone levels in HCs were within the normal reference ranges (FT3: 3.0–5.5 pmol/L; FT4: 10.2–20.7 pmol/L; TSH: 0.3–5.25 mIU/ml). The duration of OH was estimated based on the reported onset of symptoms, including fatigue, somnolence, cold intolerance, psychomotor and cognitive slowing (e.g., slowed thinking, inattention, memory impairment, and delayed responses), and affective symptoms (e.g., low mood and depressive tendencies).

Cognitive function was assessed using the Beijing version of the Montreal Cognitive Assessment (MoCA), covering visuospatial and executive function, naming, attention, language, abstraction, memory (delayed recall), and orientation. Emotional symptoms were evaluated using the 24-item Hamilton Depression Rating Scale (HAMD) and the Hamilton Anxiety Rating Scale (HAMA). All assessments were administered by trained clinicians under the supervision of experienced neurologists.

### 2.3 MRI acquisition and image preprocessing

Brain MRI examinations were performed on a 3.0-T scanner (SIGNA Architect, GE HealthCare, USA) equipped with a 20-channel head–neck coil. During scanning, participants were instructed to keep their eyes closed, remain relaxed, and stay awake. Conventional MRI sequences, including axial T1-weighted imaging (T1WI), T2-weighted imaging (T2WI), and T2-FLAIR, were performed to exclude anatomical abnormalities. DTI data were acquired using a single-shot echo-planar imaging (EPI) sequence with the following parameters: TR = 8000 ms, TE = 85 ms, slice thickness = 2 mm, number of slices = 60, matrix = 256 × 256, field of view (FOV) = 224 × 224 mm², flip angle = 90°, and diffusion weighting (b-values) of 0 and 1000 s/mm² with 32 diffusion directions. The total acquisition time was 4 min 48 s. High-resolution three-dimensional T1-weighted (3D T1) structural images of the whole brain were acquired with the following parameters: TR = 8.4 ms, TE = 3.1 ms, slice thickness = 1 mm, matrix = 256 × 256, FOV = 256 × 256 mm², FA = 8°, number of slices = 160, and acquisition time = 5 min 14 s. rs-fMRI based on blood-oxygen-level-dependent (BOLD) contrast was performed using a standard functional MRI protocol with gradient-echo and echo-planar imaging techniques. The imaging parameters were as follows: TR = 2000 ms, TE = 30 ms, slice thickness = 3 mm, FOV = 216 × 216 mm², matrix size = 72 × 72, FA = 90°, number of slices = 45, and total acquisition time = 8 min 08 s.

Preprocessing of rs-fMRI data was performed using the DPARSF v4.0 toolbox (https://www.rfmri.org/dpabi). The main steps included removal of the first 10 time points, slice-timing correction, head-motion correction, normalization to the Montreal Neurological Institute space, spatial smoothing with a 6-mm full width at half maximum Gaussian kernel, linear detrending, and regression of nuisance signals (including global signal, cerebrospinal fluid, white matter signals, and the Friston-24 head-motion parameters). A band-pass filter (0.01–0.1 Hz) was applied. Participants with head translation > 2 mm, rotation > 2°, or mean framewise displacement > 0.5 mm were excluded.

DTI data were preprocessed and analyzed using the PANDA toolbox (v1.3.1, http://www.nitrc.org/projects/panda). Eddy current distortions and head motion were corrected using rigid-body transformation of b0 images. Diffusion tensors were estimated using a linear least-squares method.

### 2.4 Construction of structural and functional networks

To construct the functional and structural networks, the brain was parcellated into 90 regions of interest (ROIs) using the Automated Anatomical Labeling 90 (AAL90) atlas. These ROIs served as the nodes of both functional and structural networks, which were constructed using weighted edges. The AAL90 parcellation was chosen because it provides an optimal balance between anatomical validity and network stability across spatial scales, as demonstrated in previous connectome studies (22).

For the construction of structural networks, white matter fiber reconstruction was performed using a deterministic fiber-tracking algorithm based on fiber assignment by continuous tracking. Tracking was terminated when fractional anisotropy (FA) <0.2 or turning angle >45°. A structural connection was defined when at least three fibers connected two regions. Edge weight was defined as the mean FA of all connecting fibers, reflecting white matter integrity. A 90 × 90 symmetric matrix was generated for each participant.

For the construction of functional networks, the mean fMRI time series was extracted from each node. Whole-brain functional networks were then constructed by calculating the Pearson correlation coefficients between the time series of all node pairs. Consistent with previous studies, negative correlations in the FC matrices were set to zero, as they may reflect spurious anti-correlations introduced by physiological noise (23, 24).

### 2.5 Graph theory analysis

G Graph theory analysis was conducted with the GRETNA toolbox. BrainNet Viewer was used for the visualization of different brain regions. Global (network efficiency and small-world attributes) and nodal topological metrics were computed for each participant. The global network efficiency was quantified using global efficiency (Eg), while small-world characteristics were assessed using five metrics: clustering coefficient (Cp), characteristic path length (Lp), normalized clustering coefficient (Gamma), normalized characteristic path length (Lambda), and small-worldness (Sigma = Gamma/Lambda). Nodal topological metrics included betweenness centrality (BC), degree centrality (DC), nodal efficiency (NE), local nodal efficiency (NLE), and nodal clustering coefficient. For functional networks, sparsity thresholds ranging from 0.05 to 0.5 (step = 0.01) were applied to ensure stable small-world estimation while reducing spurious connections.

### 2.6 SC-FC coupling

**Figure 1:**
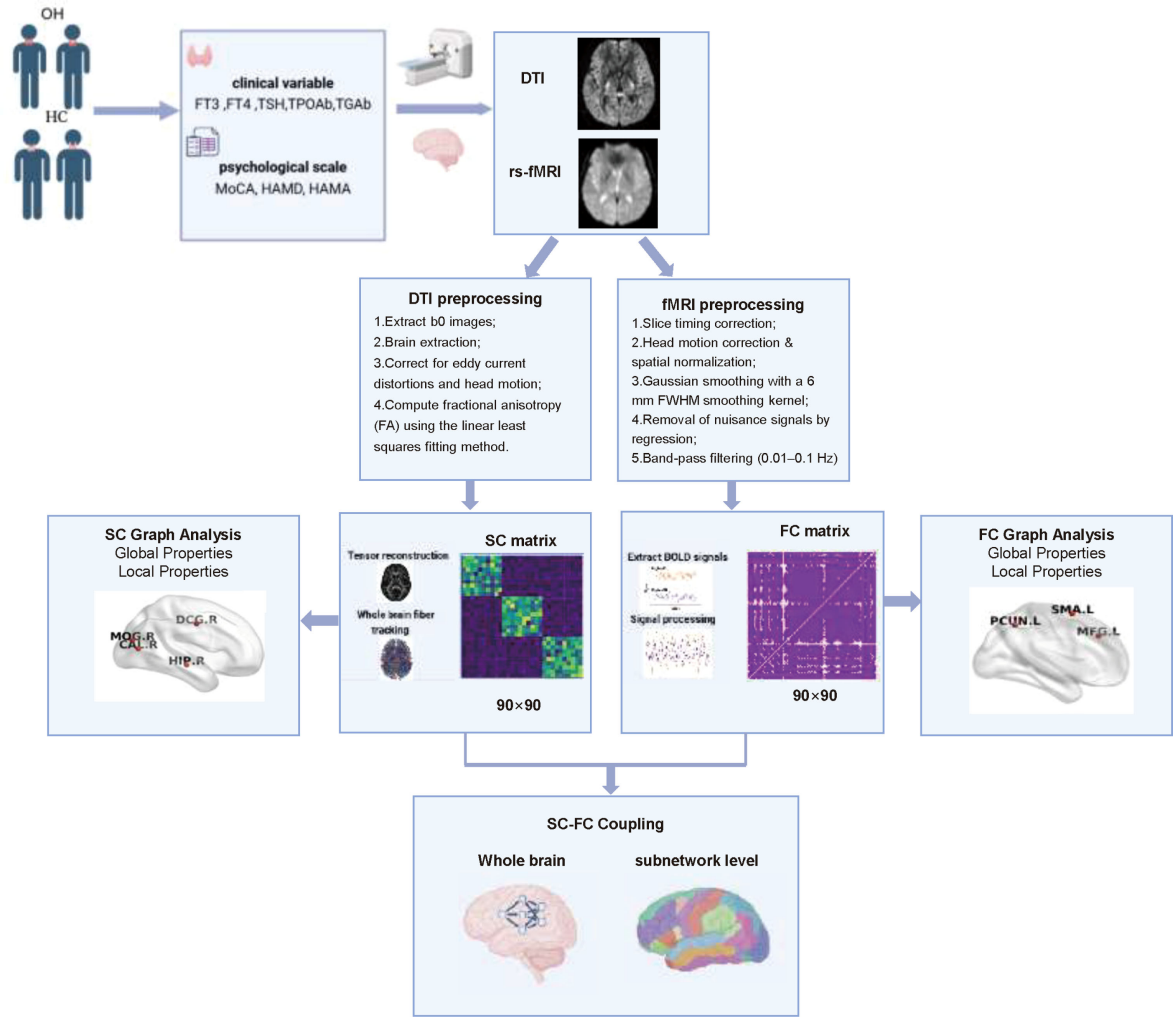
Research flowchart. HC, healthy control; OH, overt hypothyroidism. TSH, thyroid-stimulating hormone; FT3, free triiodothyronine; FT4, free thyroxine; TgAb, anti-thyroglobulin antibody; TPOAb, thyroid peroxidase antibody; MoCA, Montreal Cognitive Assessment; HAMD, Hamilton Depression Rating Scale; HAMA, Hamilton Anxiety Rating Scale.

To quantify SC-FC coupling, Pearson correlation coefficients were calculated between the SC and FC matrices for each participant. Specifically, non-zero elements in the SC matrix were first extracted as structural connections (edges) (25). Among these edges, only those with positive FC values in the corresponding FC matrix were retained. The selected SC and FC values were then vectorized into two arrays, and the Pearson correlation coefficient between these vectors was computed to obtain an individual-level whole-brain SC-FC coupling measure. This measure was subsequently used for between-group comparisons and correlation analyses with clinical variables. The data analysis pipeline is illustrated in **Figure 1**.

In addition to whole-brain SC-FC coupling analysis, this study further examined the correspondence between SC and FC matrices at the subnetwork level across distinct brain networks. Based on the Yeo-7 template, AAL90 brain regions were mapped to seven cortical networks (visual network, somatomotor network, dorsal attention network, salience network (SN), limbic system, frontoparietal network, and DMN) (21), with subcortical structures treated as a separate category. For each participant and within each predefined network, all edges connecting pairs of nodes were extracted. The SC and FC weights corresponding to these intra-network edges were then used to form two separate vectors. Subsequently, the Pearson correlation coefficient between these two vectors was calculated as the SC-FC coupling value for that participant within the specific network.

### 2.7 Statistical analysis

Statistical analyses of demographic, clinical, and psychometric variables were performed using SPSS (version 26.0). Normally distributed continuous variables are reported as mean ± SD and were compared using independent-samples t-tests; non-normally distributed variables are reported as median (P25, P75) and were compared using the Mann–Whitney U test. Categorical variables were compared using χ² tests. All tests were two-tailed, with *P* < 0.05 considered statistically significant. Group differences in global and nodal graph-theoretical metrics were assessed using a general linear model implemented in GRETNA, with group as a fixed factor. For structural networks, sex, age, and years of education were included as covariates, whereas for functional networks, mean FD was additionally included to control for head motion. Multiple comparisons across nodal and network-level metrics were controlled using the false discovery rate (FDR), with *P*_FDR_ < 0.05 considered statistically significant. Between-group differences in SC-FC coupling were examined using analysis of covariance (ANCOVA), controlling for sex, age, and years of education. Multiple comparisons were likewise corrected using FDR (*P*_FDR_ < 0.05). Partial Pearson correlation analyses were performed to examine the associations between measures showing significant between-group differences and clinical variables, controlling for sex, age, and education. Multiple comparisons in correlation analyses were additionally controlled using FDR (*P*_FDR_ < 0.05).

## 3 Results

### 3.1 Demographic and clinical characteristics

A total of 100 participants were included, comprising 45 patients with OH (27 females and 18 males; mean age: 47.33 ± 10.45 years) and 55 HCs (35 females and 20 males; mean age: 44.73 ± 10.79 years). There were no significant between-group differences in age, sex, BMI, or years of education. Compared with HCs, patients with OH showed significantly lower MoCA scores and higher HAMD and HAMA scores (*P* < 0.05; **Table 1**). In addition, no significant group differences were observed in rs-fMRI head motion parameters, including maximum translation and rotation along the X, Y, and Z axes, as well as mean FD_Jenkinson (*P* > 0.05; **Table 2**).

**Table 1:** Demographic characteristics, clinical variables, and neuropsychological scores in patients with overt hypothyroidism and healthy controls.

Variables	OH (*n* = 45)	HC (*n* = 55)	*χ^2^*/t/z value	*P-*value
Demographic				
Age (years)	47.33 ± 10.45	44.73 ± 10.79	1.218	0.226
Sex (*n* [%])			0.139	0.709
Female	27 (60.0%)	35 (63.6%)		
Male	18 (40.0%)	20 (36.4%)		
BMI (kg/m^2^)	23.43 ± 1.88	24.11 ± 2.96	-1.340	0.183
Education (years)	12 (9, 16)	12 (9, 16)	-0.241	0.810
Smoking status			0.732	0.392
Yes	25 (55.6%)	34 (61.8%)		
No	20 (44.4%)	21 (38.2%)		
Drinking			1.217	0.270
Yes	14 (31.1%)	23 (41.8%)		
No	31 (68.9%)	32 (58.2%)		
Clinical characteristic				
Duration (months)	9.16 ± 0.78	-	-	-
FT3 (pmol/L)	3.12 (2.65, 3.29)	5.10 (4.30, 5.66)	−8.791	< 0.001
FT4 (pmol/L)	8.78 (8.25, 9.35)	15.09 (13.10, 17.98)	−8.760	< 0.001
TSH (mIU/L)	64.31 (45.26, 89.21)	1.69 (1.15, 2.50)	−8.580	< 0.001
TPOAb (IU/ml)	3188.96 ± 159.49	-	-	-
TGAb (IU/ml)	3486.71 ± 158.09	-	-	-
Neuropsychological tests				
MoCA	26.00 (24.00, 28.00)	28.00 (27.00, 28.00)	−4.091	< 0.001
HAMD	4.00 (2.00, 7.00)	2.00 (1.00, 4.00)	−3.554	<0.001
HAMA	13.00 (8.00, 17.00)	2.00 (1.00, 2.00)	−7.660	< 0.001

HC, healthy controls; OH, overt hypothyroidism; TSH, thyroid-stimulating hormone; FT3, free triiodothyronine; FT4, free thyroxine; TgAb, anti-thyroglobulin antibodies; TPOAb, thyroid peroxidase antibodies. MoCA, Montreal Cognitive Assessment; HAMD, Hamilton Rating Scale for Depression-24; HAMA, Hamilton Anxiety Rating Scale.

**Table 2:** For the comparison of group differences in the head motion parameters.

Metric	OH (*n* = 45)	HC (*n* = 55)	*P-*value
max Translation in X axis (mm)	0.264 ± 0.139	0.283 ± 0.255	0.652
max Translation in Y axis (mm)	0.933 ± 0.574	0.887 ± 0.599	0.698
max Translation in Z axis (mm)	0.710 ± 0.406	0.640 ± 0.399	0.390
max Rotation in X axis (degree)	0.662 ± 0.452	0.655 ± 0.434	0.937
max Rotation in Y axis (degree)	0.480 ± 0.289	0.375 ± 0.370	0.125
max Rotation in Z axis (degree)	0.388 ± 0.317	0.377 ± 0.514	0.899
mean FD_Jenkinson	0.095 ± 0.060	0.085 ± 0.045	0.385

### 3.2 Global topological properties of brain networks in OH patients

**Figure 2:**
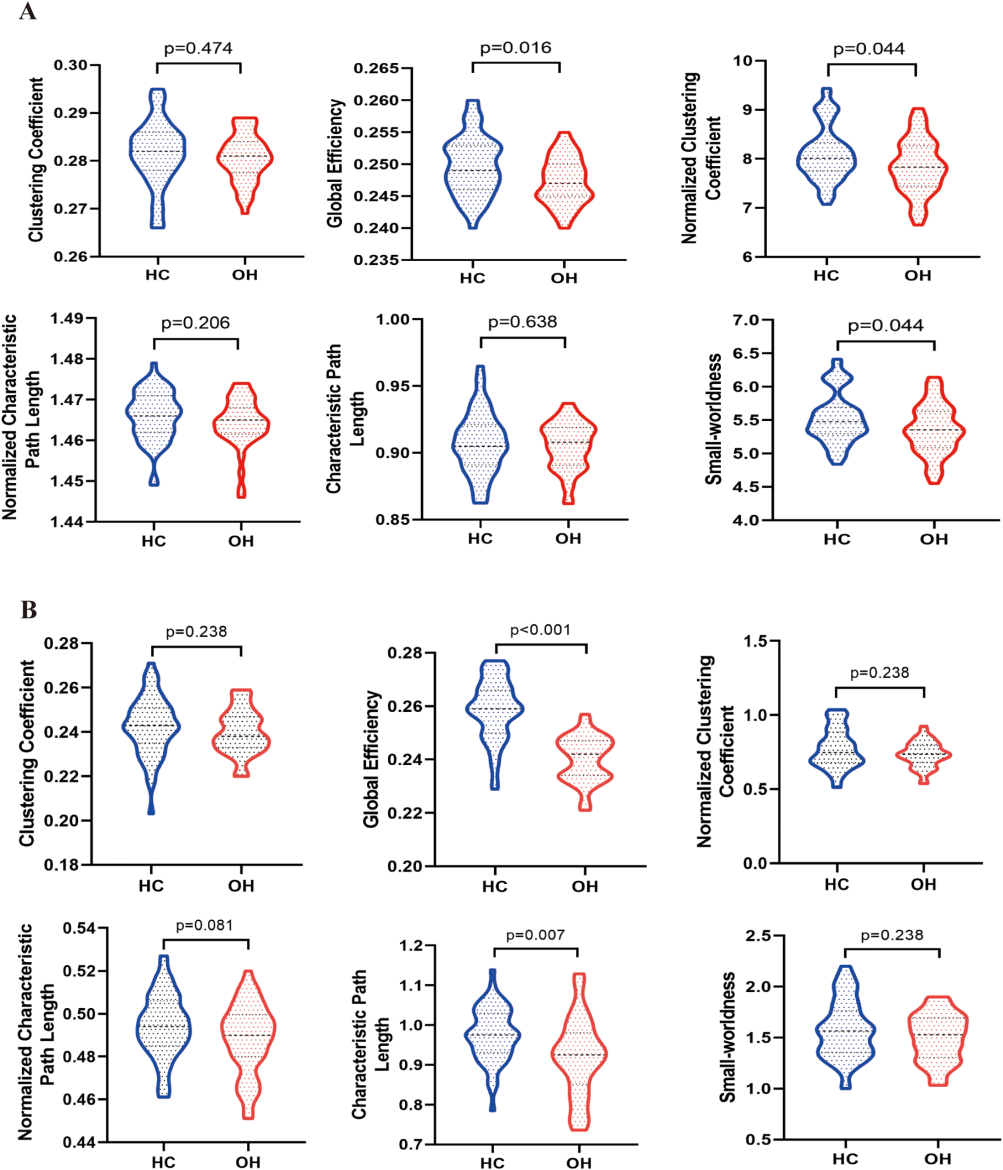
Global topological properties in OH patients and HC. **(A)** Global topological properties of the structural network in OH patients and HC. **(B)** Global topological properties of the functional network in OH patients and HC. The labeled *p*-values are FDR-corrected. Analyses of the structural network were adjusted for sex, age, and years of education, whereas analyses of the functional network were additionally adjusted for mean FD. HC, healthy control; OH, overt hypothyroidism.

Both the structural and functional networks of the OH and HC groups exhibited small-world characteristics (Sigma > 1). Compared with HCs, the OH group showed a significantly lower normalized clustering coefficient (Gamma) (*P*_FDR_ = 0.044) and small-worldness (Sigma) (*P*_FDR_ = 0.044) in the structural network. In the functional network, the OH group exhibited a shorter Lp (*P*_FDR_ = 0.007). Eg was reduced in both SC and FC networks in OH patients (SC: *P*_FDR_ = 0.016; FC: *P*_FDR_ < 0.001), as illustrated in **Figure 2**.

### 3.3 Nodal topological properties of brain networks in OH patients

**Figure 3:**
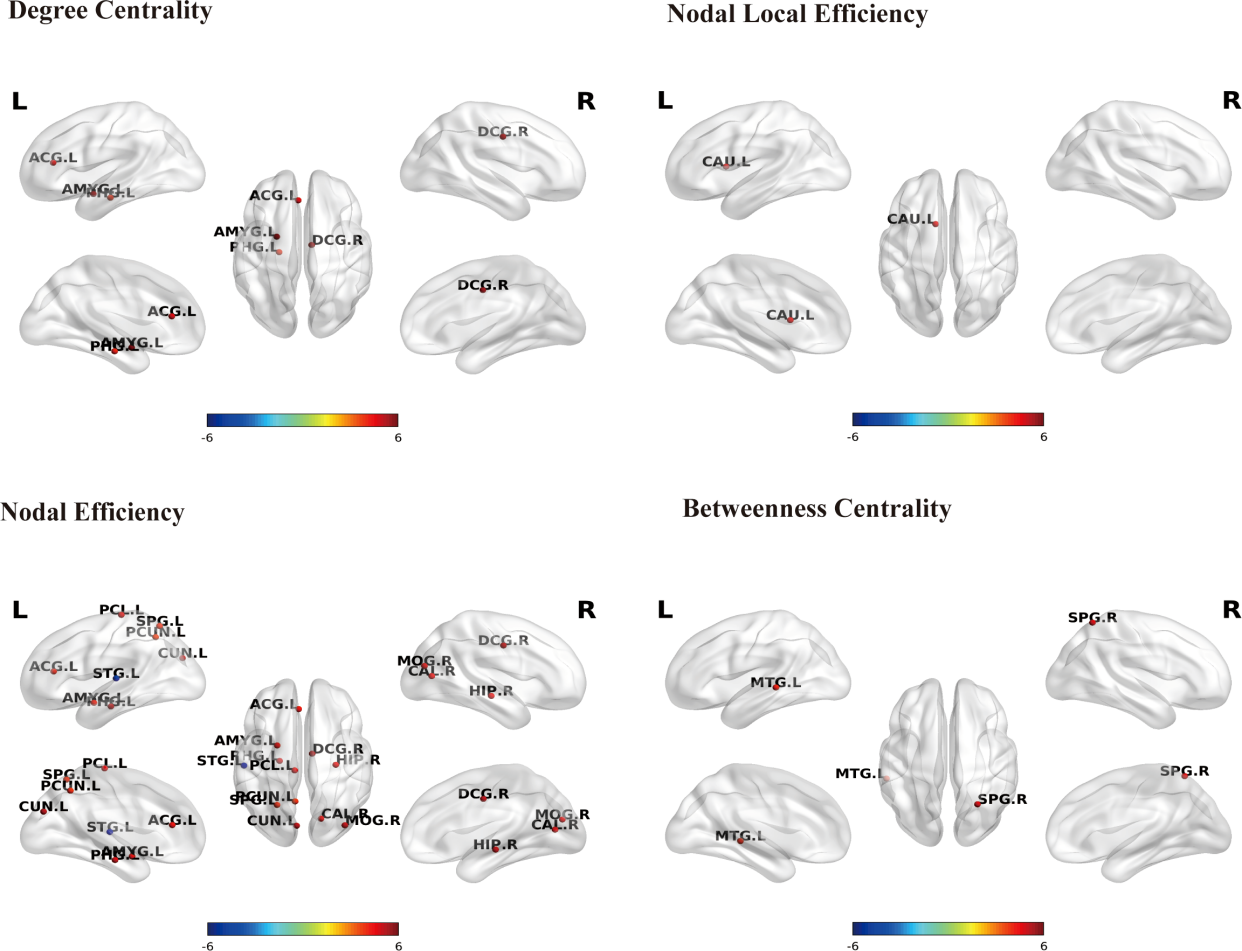
Brain regions showing significant between-group differences in nodal topological metrics within the structural connectivity network. Multiple comparisons were corrected using the FDR method (P_FDR_ < 0.01). Nodes are labeled according to the AAL90 atlas (L/R indicate left/right hemisphere). Warm colors indicate decreased nodal properties in the OH group compared with HC, whereas cool colors indicate increased nodal properties in the OH group relative to HC. Darker colors represent greater statistical differences between groups. ACG, anterior cingulate gyrus; AMYG, amygdala; CAL, calcarine cortex; CAU, caudate; CUN, cuneus; DCG, middle cingulate gyrus; HIP, hippocampus; MTG, middle temporal gyrus; MOG, middle occipital gyrus; PCL, paracentral lobule; PCUN, precuneus; PHG, parahippocampal gyrus; SPG, superior parietal gyrus; STG, superior temporal gyrus.

Graph‐theoretical analysis revealed widespread alterations in regional nodal properties in OH patients, with predominantly decreased metrics. Specifically, in the structural network, NE was reduced across multiple regions, including temporal/limbic structures such as the parahippocampal gyrus (PHG.L), amygdala (AMYG.L), and hippocampus (HIP.R); cingulate cortex regions (DCG.R and ACG.L); parietal areas (SPG.L and PCUN.L); occipital regions (CUN.L, MOG.R, and CAL.R); and the medial and the medial sensorimotor cortex (PCL.L), whereas STG.L showed increased structural NE. In addition, BC was markedly reduced in SPG.R and MTG.L, while DC was significantly diminished in PHG.L, AMYG.L, ACG.L, and DCG.R. A decrease in NLE was also observed in CAU.L (**Figure 3** and **Table 3**). Overall, patients with OH exhibited topological abnormalities at the structural network level, primarily characterized by decreased nodal efficiency and centrality metrics, mainly involving the limbic system, cingulate cortex, and frontoparietal-related brain regions.

**Table 3:** Brain regions showing significant differences in nodal topological properties between the OH group and HC.

Graph measures	Brain region (AAL90)	Anatomical category	t-value	*PFDR* value
Structural network				
Betweenness centrality				
	SPG.R	Parietal Lobe	4.764	5.92E-04
	MTG.L	Temporal Lobe	4.130	3.44E-03
Degree centrality				
	PHG.L	Temporal Lobe	4.101	1.91E-03
	AMYG.L	Temporal Lobe	8.275	5.90E-11
	DCG.R	Cingulate/Limbic	5.526	1.22E-05
	ACG.L	Cingulate/Limbic	4.812	1.63E-04
Nodal efficiency				
	CUN.L	Occipital Lobe	5.304	4.78E-05
	DCG.R	Cingulate/Limbic	5.206	4.78E-05
	STG.L	Temporal Lobe	-4.881	1.13E-04
	PCL.L	Frontal/Parietal Lobe	4.807	1.13E-04
	PHG.L	Temporal Lobe	4.765	1.13E-04
	HIP.R	Temporal Lobe	4.709	1.13E-04
	MOG.R	Occipital Lobe	4.691	1.13E-04
	CAL.R	Occipital Lobe	4.502	2.09E-04
	ACG.L	Cingulate/Limbic	4.361	3.20E-04
	AMYG.L	Temporal Lobe	4.214	5.03E-04
	SPG.L	Parietal Lobe	4.052	8.32E-04
	PCUN.L	Parietal Lobe	3.983	9.81E-04
Nodal local efficiency				
	CAU.L	Subcortical	4.896	3.47E-04
Functional network				
Degree centrality				
	DCG.L	Cingulate/Limbic	5.039	1.925E-04
	AMYG.L	Temporal Lobe	4.458	9.920E-04
	PHG.L	Temporal Lobe	3.465	2.367E-03
Nodal efficiency				
	PCG.R	Cingulate/Limbic	5.989	3.145E-06
	MFG.L	Frontal Lobe	5.155	5.944E-05
	SMA.L	Frontal Lobe	4.400	8.257E-04
	PHG.R	Temporal Lobe	-4.032	2.418E-03
	PCUN.L	Parietal Lobe	3.976	2.418E-03

*P*_FDR_ represents the *P*-value after false discovery rate (FDR) correction (*P*_FDR_ < 0.01). The t-value reflects the statistical difference between the overt hypothyroidism (OH) group and healthy controls (HCs). A positive t value indicates that the OH group shows lower nodal topological properties compared with HCs. ACG, anterior cingulate gyrus; AMYG, amygdala; CAL, calcarine cortex; CAU, caudate; CUN, cuneus; DCG, middle cingulate gyrus; HIP, hippocampus; MTG, middle temporal gyrus; PCG, posterior cingulate gyrus; PCL, paracentral lobule; PCUN, precuneus; PHG, parahippocampal gyrus; SMA, supplementary motor area; SPG, superior parietal gyrus; STG, superior temporal gyrus. HC, healthy control; OH, overt hypothyroidism.

**Figure 4:**
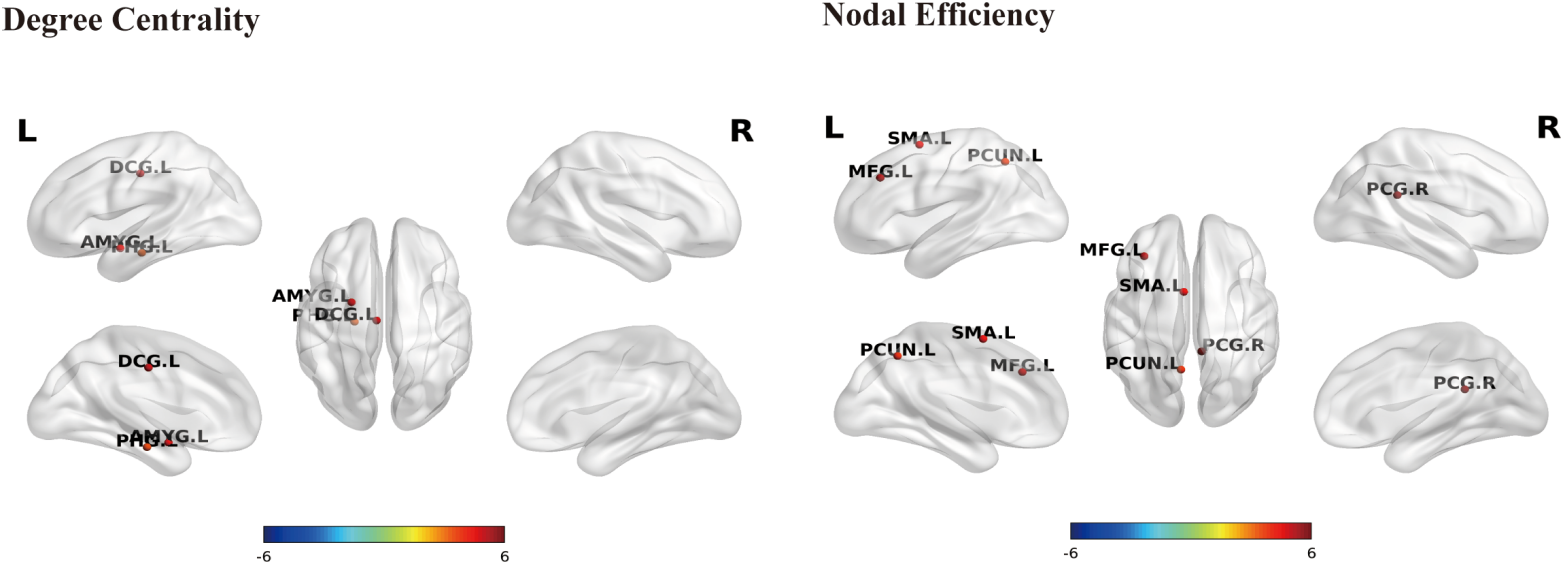
Brain regions showing significant between-group differences in nodal topological metrics within the functional connectivity network. Multiple comparisons were corrected using the FDR method (*P*_FDR_ < 0.01). Nodes are labeled according to the AAL90 atlas (L/R indicate left/right hemisphere). Warm colors indicate decreased nodal properties in the OH group compared with HC, whereas cool colors indicate increased nodal properties in the OH group relative to HC. Darker colors represent greater statistical differences between groups. AMYG, amygdala; DCG, middle cingulate gyrus; MFG, middle frontal gyrus; PCG, posterior cingulate gyrus; PCUN, precuneus; PHG, parahippocampal gyrus; SMA, supplementary motor area.

Functional network analysis further revealed abnormal patterns in nodal centrality and efficiency. Reductions in DC were predominantly localized to limbic/cingulate regions, including AMYG.L, DCG.L, and PHG.L. In contrast, decreases in NE were observed in PCG.R, supplementary motor area (SMA.L), middle frontal gyrus (MFG.L), and PCUN.L. PHG.R exhibited increased functional NE (**Figure 4** and **Table 3**). Overall, patients with OH also exhibited an abnormal pattern at the functional network level, primarily characterized by decreased nodal topological metrics, with these changes mainly concentrated in the limbic system and frontoparietal-related functional regions.

### 3.4 Abnormal SC-FC coupling

**Figure 5:**
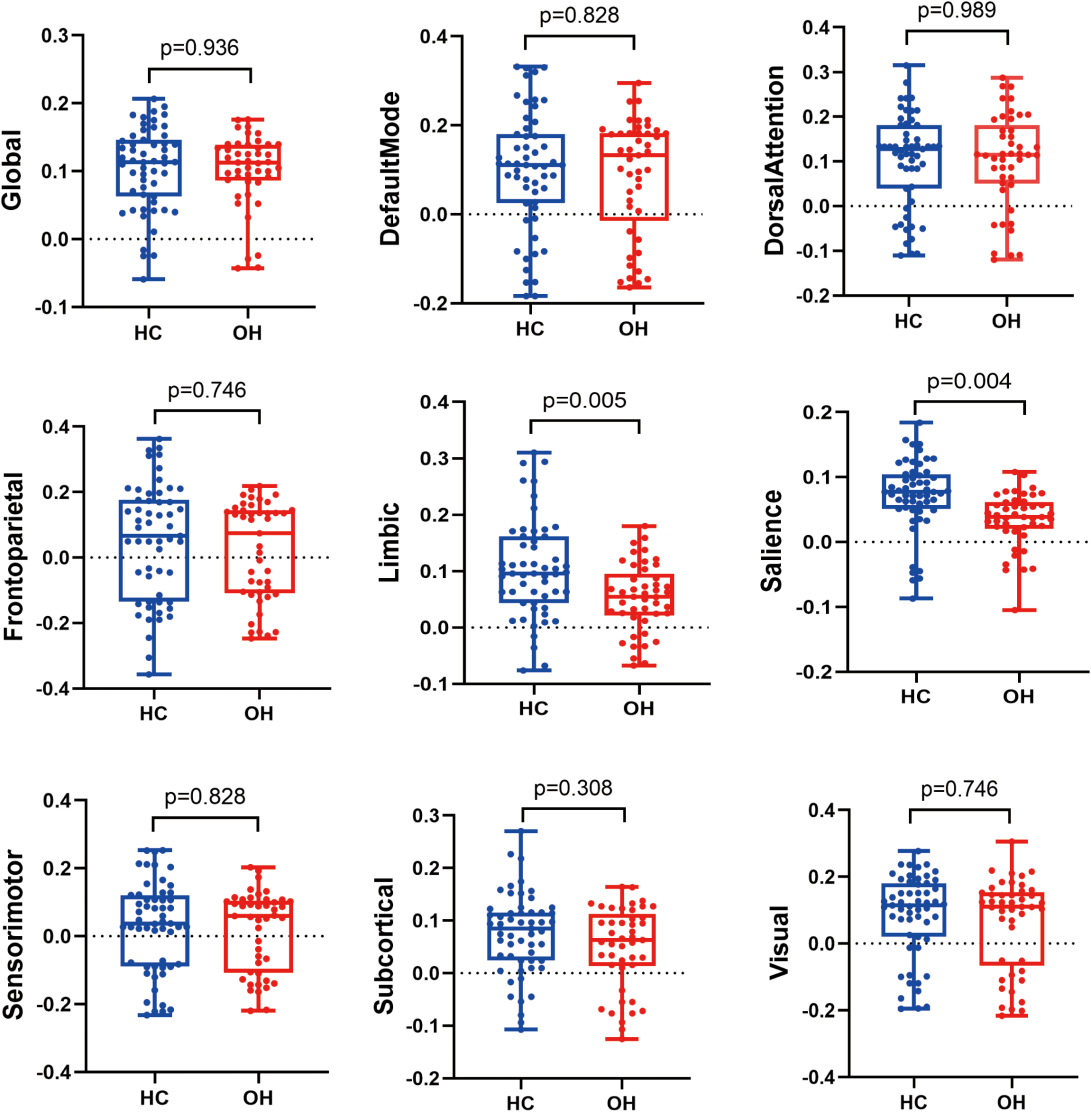
Comparison of SC-FC coupling at the whole-brain level and across eight subnetworks between HC and OH groups. Group differences were assessed using analysis of covariance (ANCOVA), controlling for sex, age, and years of education. *P*-values shown are FDR-corrected. HC, healthy control; OH, overt hypothyroidism.

At the whole-brain level, global SC-FC coupling did not differ between the OH and HC groups (*P*_FDR_ = 0.936) (**Figure 5**). However, subnetwork level analyses revealed reduced SC-FC coupling within the limbic system (*P*_FDR_ = 0.005) and SN (*P*_FDR_ = 0.004) in OH compared with HCs.

### 3.5 Associations of network metrics with clinical and neuropsychological measures

**Figure 6:**
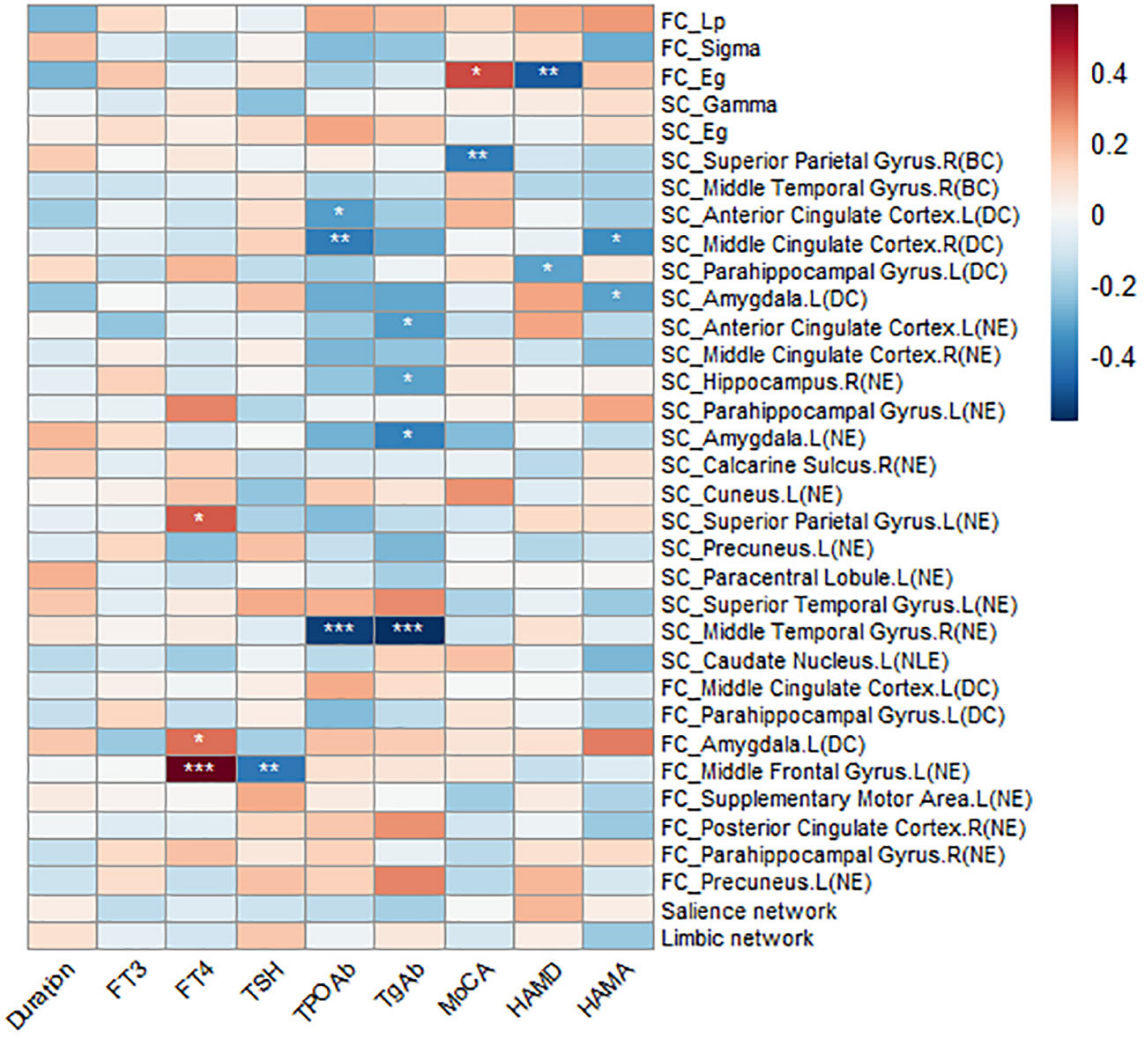
Correlation heatmap between graph metrics, subnetwork coupling, and clinical assessment scales. Blue indicates negative correlations, red indicates positive correlations, and deeper color shades represent stronger correlations. *p < 0.05, **p < 0.01, ***p < 0.001. Abbreviations: FC, functional connectivity; SC, structural connectivity; Lp, characteristic path length; Gamma, normalized clustering coefficient; Sigma, small-worldness; Eg, global efficiency; BC, betweenness centrality; DC, degree centrality; NE, nodal efficiency; NLE, nodal local efficiency.

Partial correlation analyses (controlling for sex, age, and years of education) were performed to examine the associations between between-group difference indicators and clinical variables, as well as psychological scale scores (**Figure 6**). No significant correlations were observed between SC-FC coupling and clinical or psychological measures. Regarding topological metrics (**Table 4**), at the functional network level, FT4 levels were positively correlated with the NE of the left middle frontal gyrus (*r* = 0.585, *P*_FDR_ = 0.002). In contrast, at the structural network level, thyroid autoantibodies (TPOAb and TgAb) were negatively correlated with the NE of the right middle temporal gyrus (TPOAb: *r* = −0.550, *P*_FDR_ = 0.006; TgAb: *r* = −0.588, *P*_FDR_ = 0.001). These findings suggest that thyroid hormone levels and autoimmune status may be differentially associated with regional information transfer efficiency. Furthermore, HAMD scores were negatively correlated with the Eg of the functional network (*r* = −0.487, *P*_FDR_ = 0.036), suggesting that reduced network efficiency may underlie depressive symptoms in patients with OH.

**Table 4:** Significant correlations between altered nodal topological metrics and clinical/neuropsychological measures in the OH group.

Variables	Topological metrics [brain region (metric)]	*r*	*P*-value	*P_FDR_*value
SC network				
FT4	left superior parietal gyrus (NE)	0.359	0.019	0.331
TPOAb	right middle temporal gyrus (NE)	−0.550	< 0.001	0.006
	right middle cingulate cortex (DC)	−0.408	0.007	0.126
	left anterior cingulate cortex (DC)	−0.318	0.040	0.407
TgAb	right middle temporal gyrus (NE)	−0.588	< 0.001	0.001
	left amygdala (NE)	−0.396	0.010	0.162
	left anterior cingulate cortex (NE)	−0.321	0.038	0.269
	right hippocampus (NE)	−0.311	0.045	0.269
HAMD	left parahippocampal gyrus (DC)	−0.312	0.044	0.752
HAMA	right middle cingulate cortex (DC)	−0.362	0.018	0.446
	left amygdala (DC)	−0.311	0.045	0.446
FC network				
FT4	left middle frontal gyrus (NE)	0.585	< 0.001	0.002
	left amygdala (DC)	0.321	0.038	0.433
TSH	left middle frontal gyrus (NE)	−0.405	0.008	0.263
MoCA	Eg	0.387	0.011	0.383
HAMD	Eg	−0.487	0.001	0.036

Partial correlations were computed within the OH group, adjusting for sex, age, and years of education. P_FDR_ represents the P value after false discovery rate (FDR) correction. FT4, free thyroxine; TgAb, anti-thyroglobulin antibody; TPOAb, thyroid peroxidase antibody; MoCA, Montreal Cognitive Assessment; HAMD, 24-item Hamilton Depression Rating Scale; HAMA, Hamilton Anxiety Rating Scale; SC, structural connectivity network; FC, functional connectivity network; Eg, global efficiency; NE, nodal efficiency; DC, degree centrality.

## 4 Discussion

This multimodal study revealed disrupted brain network topology and SC-FC coupling in patients with OH. At the global level, reduced small-worldness in the structural network, together with decreased global efficiency in both structural and functional networks, indicates a less efficient balance between network segregation and integration. At the regional level, nodal abnormalities involved several nodes with altered topological properties, suggesting focal vulnerability within systems supporting cognitive and affective functions. Notably, subnetwork level analyses revealed selectively reduced SC-FC coupling within the limbic and salience networks. Such precise disconnection between structural architecture and functional dynamics, together with its significant association with clinical symptoms, provides a novel neurobiological explanation for the core neuropsychiatric manifestations of hypothyroidism.

### 4.1 Global topological properties of structural and functional networks in OH patients

Although small-world organization was preserved, patients with OH showed significant alterations in global network topology. In the structural network, reduced Gamma and Sigma indicate a less optimal small-world configuration, reflecting an altered balance between local clustering and global integration (26). Consistently, the observed decrease in Eg reflects a decline in the overall capacity of the OH brain network to integrate information across distant regions (27). The Lp represents the average number of steps required for information transfer within the network (28). Regular networks are characterized by tightly clustered local connections, resulting in high Cp and long Lp, whereas random networks exhibit disordered connections with lower Cp but shorter Lp (29). Notably, a shorter Lp should not be interpreted as improved communication efficiency; rather, when considered alongside reduced Eg and reduced structural small-worldness, these findings may be compatible with a more random-like reconfiguration of large-scale network organization in OH patients. Furthermore, the positive correlation between MoCA scores and Eg, along with the negative correlation between HAMD scores and Eg, suggests that reduced network efficiency may underlie the cognitive impairments and depressive symptoms observed in OH patients. Despite these abnormalities, the overall small-world property was preserved, implying potential compensatory reorganization that helps maintain basic neural communication. Similar compensatory mechanisms have been reported in other cognitive and affective disorders (30–32).

### 4.2 Local topological properties of structural and functional networks in OH patients

Nodal analyses revealed widespread alterations in efficiency and centrality across higher order brain systems, including the limbic system, DMN, and SN. Within the limbic system, core regions such as the hippocampus, parahippocampal gyrus, amygdala, and cingulate cortex exhibited significant alterations in NE and DC, predominantly showing reduced values. Notably, these regional abnormalities were significantly associated with thyroid autoantibody and hormone levels, suggesting that the limbic system may represent a key neural substrate underlying cognitive and emotional disturbances in OH. Previous neuroimaging studies have reported structural damage in limbic regions in OH (6, 8, 33), and our findings provide complementary network-level evidence. Clinical correlation analyses further showed that the positive association between cognitive performance and Eg of the functional network may represent a direct neural correlate of cognitive impairment in hypothyroidism. In addition, accumulating evidence indicates that limbic dysfunction is a hallmark of progression from mild cognitive impairment to Alzheimer’s disease (34, 35). Together, these findings suggest that limbic circuitry may represent a shared locus of vulnerability for both cognitive and emotional dysfunction in OH.

Furthermore, decreased nodal efficiency was observed in key DMN regions, including the posterior cingulate cortex, precuneus, and superior parietal gyrus. These findings suggest impaired information integration both within the DMN and between the DMN and other large-scale networks. These regions are critically involved in higher-order cognitive processes such as self-referential processing, emotional regulation, and memory consolidation (36, 37). Consistent with our results, DMN abnormalities have been widely reported in neuropsychiatric and neurodegenerative disorders, including Alzheimer’s disease (38), Parkinson’s disease (39), major depressive disorder (40), and other cognitive or affective disorders (41). Recent studies of white matter network topology have also demonstrated localized disruptions within the limbic and DMN subsystems in hypothyroidism (15), further supporting the role of impaired network integration in the neurocognitive and emotional symptoms of the disease.

In addition, abnormalities were observed in key nodes of the SN, particularly the anterior cingulate cortex, suggesting impaired conflict monitoring and resource allocation in response to internal and external stimuli. The SN plays a central role in detecting salient stimuli and coordinating interactions among large-scale brain networks, thereby integrating emotional and sensory information (42). Alterations in the SN have been consistently reported across multiple neuropsychiatric disorders and may serve as potential biomarkers of disease progression (42–44).

Overall, OH is associated with disruptions in nodal topology within higher-order functional networks, including the limbic system, DMN, and SN. These disruptions are reflected not only in reduced nodal influence (e.g., decreased DC and NE), but also in diminished capacity for global integration and local resilience. Collectively, these alterations may represent a network-level substrate underlying cognitive and emotional deficits in hypothyroidism.

### 4.3 SC-FC coupling in OH patients

SC-FC coupling, which reflects the pairwise correspondence between SC and FC, has been widely used to investigate disease-related alterations in neural systems (45, 46). At the whole-brain level, we observed no significant group differences, suggesting that global SC-FC coupling may be relatively preserved in OH. However, subnetwork analyses revealed a more specific pattern, with selectively reduced coupling within the limbic system and the SN, two subsystems critically involved in emotion and cognition. Aberrant SC-FC coupling may arise from divergent changes between structural and functional networks (47). In early-stage pathological conditions, structural alterations may be accompanied by functional reorganization that helps maintain interregional communication, potentially weakening structure–function correspondence at the subnetwork level (48, 49). In this context, subnetwork-level analyses may be more sensitive than whole-brain metrics. Previous studies have highlighted that regional SC-FC coupling is a distinctive feature of brain organization, exhibiting substantial variability across regions and being influenced by genetic factors, highlighting the utility of subnetwork-level analyses (50). In our study, reduced coupling within the limbic system is consistent with prior reports of structural and functional impairment in OH patients (6, 15). Similarly, SC-FC coupling within the SN was decreased. The SN plays a pivotal role in cognitive control by coordinating the allocation of cortical resources and prioritizing information processing (51, 52), and has been implicated in the pathophysiology of depression (53). Taken together, these module-specific reductions in SC-FC coupling provide a network-level account of altered structure–function correspondence in OH, complementing the observed associations between network metrics and clinical symptom severity. SC-FC coupling primarily reflects the anatomical constraints imposed by white matter on FC. However, accumulating evidence suggests that white matter regions can also exhibit detectable BOLD signals and form functionally coherent patterns, offering additional insights into structure–function relationships (54). Future studies integrating white matter FC may provide a more comprehensive understanding of the mechanisms underlying SC-FC coupling.

### 4.4 Limitations

This study has several limitations. First, the single-center, cross-sectional design with a modest sample size limits generalizability and precludes causal inference regarding the relationship between OH and brain network alterations or their longitudinal changes. Second, structural networks were derived from DTI-based tractography, which is limited in resolving complex fiber architecture and may affect the accuracy of specific connections. Although head motion was controlled using exclusion criteria, residual motion and physiological noise may still influence resting-state measures. Third, connectome metrics may be affected by the choice of brain parcellation schemes and spatial resolution (55); future studies should validate the robustness of the findings across different atlases, thresholds, and network construction strategies. Finally, SC-FC coupling was defined based on direct structure–function correspondence, without accounting for indirect pathways, which may also contribute to functional organization (56). In addition, future studies should apply the proposed analytical framework to multi-center and internationally diverse datasets to further validate the robustness and generalizability of the findings.

## 5 Conclusion

OH was associated with convergent abnormalities in brain network topology and structure-function coupling. OH patients showed altered global organization, widespread nodal disruptions involving higher order systems, and selective reductions of SC-FC coupling within the limbic and salience networks. These network-level alterations were linked to clinical and neuropsychological measures, highlighting multimodal connectomic metrics as potential markers of cognitive and affective disturbances in hypothyroidism.

## Data Availability

The original contributions presented in the study are included in the article/supplementary material. Further inquiries can be directed to the corresponding author.
